# PDE4B Missense Variant Increases Susceptibility to Post-traumatic Stress Disorder-Relevant Phenotypes in Mice

**DOI:** 10.1523/JNEUROSCI.0137-24.2024

**Published:** 2024-09-10

**Authors:** Tatiana V. Lipina, Shupeng Li, Ekaterina S. Petrova, Tamara G. Amstislavskaya, Ryan T. Cameron, Christina Elliott, Yoichi Gondo, Alexander McGirr, Jonathan G. L. Mullins, George S. Baillie, James R. Woodgett, Steven J. Clapcote

**Affiliations:** ^1^Department of Pharmacology & Toxicology, University of Toronto, Toronto, Ontario M5S 1A8, Canada; ^2^School of Chemical Biology and Biotechnology, Shenzhen Graduate School, Peking University, Shenzhen 518071, China; ^3^Federal State Budgetary Scientific Institution, Scientific Research Institute of Physiology & Basic Medicine, Novosibirsk 630117, Russia; ^4^School of Cardiovascular & Metabolic Health, College of Medical, Veterinary & Life Sciences, University of Glasgow, Glasgow G12 8TA, United Kingdom; ^5^Mutagenesis and Genomics Team, RIKEN BioResource Center, Tsukuba, Ibaraki 305-0074, Japan; ^6^Hotchkiss Brain Institute, University of Calgary, Calgary, Alberta T2N 4N1, Canada; ^7^Institute of Life Science, Swansea University, Swansea SA3 5AU, United Kingdom; ^8^Lunenfeld-Tanenbaum Research Institute, Mount Sinai Hospital, Toronto, Ontario M5G 1X5, Canada; ^9^School of Biomedical Sciences, University of Leeds, Leeds LS2 9JT, United Kingdom

**Keywords:** cAMP, fear memory, PDE4B, PTSD, schizophrenia, trauma

## Abstract

Large-scale genome-wide association studies (GWASs) have associated intronic variants in *PDE4B*, encoding cAMP-specific phosphodiesterase-4B (PDE4B), with increased risk for post-traumatic stress disorder (PTSD), as well as schizophrenia and substance use disorders that are often comorbid with it. However, the pathophysiological mechanisms of genetic risk involving PDE4B are poorly understood. To examine the effects of PDE4B variation on phenotypes with translational relevance to psychiatric disorders, we focused on PDE4B missense variant M220T, which is present in the human genome as rare coding variant rs775201287. When expressed in HEK-293 cells, PDE4B1-M220T exhibited an attenuated response to a forskolin-elicited increase in the intracellular cAMP concentration. In behavioral tests, homozygous *Pde4b*^M220T^ male mice with a C57BL/6JJcl background exhibited increased reactivity to novel environments, startle hyperreactivity, prepulse inhibition deficits, altered cued fear conditioning, and enhanced spatial memory, accompanied by an increase in cAMP signaling pathway-regulated expression of BDNF in the hippocampus. In response to a traumatic event (10 tone–shock pairings), neuronal activity was decreased in the cortex but enhanced in the amygdala and hippocampus of *Pde4b*^M220T^ mice. At 24 h post-trauma, *Pde4b*^M220T^ mice exhibited increased startle hyperreactivity and decreased plasma corticosterone levels, similar to phenotypes exhibited by PTSD patients. Trauma-exposed *Pde4b*^M220T^ mice also exhibited a slower decay in freezing at 15 and 30 d post-trauma, demonstrating enhanced persistence of traumatic memories, similar to that exhibited by PTSD patients. These findings provide substantive mouse model evidence linking PDE4B variation to PTSD-relevant phenotypes and thus highlight how genetic variation of PDE4B may contribute to PTSD risk.

## Significance Statement

Human genetic studies have associated variants in the *PDE4B* gene, encoding the phosphodiesterase-4B (PDE4B) enzyme, with increased risk for post-traumatic stress disorder (PTSD) and other mental disorders that often occur with it. However, the underlying biological mechanisms of genetic risk involving PDE4B are poorly understood. To examine the effect of PDE4B variation on behaviors relevant to mental disorders, we studied male *Pde4b*^M220T^ mice that carry a PDE4B variant (M220T), which is also present in humans. *Pde4b*^M220T^ mice exhibited increased PTSD-like behavior in response to a traumatic event, as well as abnormal neuronal activity in the brain. Our findings provide substantive evidence linking PDE4B variation to PTSD-relevant behaviors and thus highlight how genetic variation of PDE4B may contribute to PTSD risk.

## Introduction

Psychiatry has lagged behind other medical fields in mechanistic understanding and the development of improved therapeutics. However, recent large-scale genome-wide association studies (GWASs) are beginning to elucidate the etiology and pathophysiology of psychiatric disorders by identifying genes containing common single nucleotide polymorphisms (SNPs) that increase the risk for disease at genome-wide significance (GWS) levels. *PDE4B*, encoding cAMP-specific phosphodiesterase-4B (PDE4B), is notable among GWS risk genes by virtue of its pleiotropic effects across diagnostic boundaries.

In a GWAS of post-traumatic stress disorder (PTSD) and other stress-related diagnoses, *PDE4B* was the only GWS risk locus identified (Meier et al., 2019). A GWAS of re-experiencing symptoms (involuntary retrieval of traumatic memories), a distinctive feature of PTSD, in military veterans identified *PDE4B* as 1 of 30 genes that reached GWS levels (Gelernter et al., 2019). In a subsequent meta-analysis of 88 PTSD GWAS datasets, *PDE4B* was among 43 GWS genes prioritized as likely causal (Nievergelt et al., 2024). Transcriptome profiling revealed that *PDE4B* mRNA expression levels in blood were lower in PTSD patients than those in controls and significantly correlated with the severity of re-experiencing symptoms and trait anxiety and with *PDE4B* DNA methylation levels (Hori et al., 2024).

The majority of individuals with PTSD have one or more comorbid psychiatric disorders (Kessler et al., 1995). A cross-trait meta-analysis of GWAS datasets identified *PDE4B* as one of five GWS risk loci shared by anxiety and stress-related diagnoses (including PTSD) and major depressive disorder (Mei et al., 2022). In a GWAS of schizophrenia, *PDE4B* was 1 of 106 protein-coding genes prioritized as likely causal (Trubetskoy et al., 2022). *PDE4B* was also among the top 3 of 42 GWS risk genes identified in a GWAS meta-analysis of general addiction risk derived from substance use disorders (Hatoum et al., 2023).

Finding loci statistically associated with increased risk of psychiatric disorders is merely the start of a long process toward meaningful biological understanding, let alone better treatments. The causal variants that drive the statistical associations, and their biological consequences, are yet to be identified, and thus the pathophysiological mechanisms of genetic risk involving *PDE4B* are poorly understood. PDE4B is one of four subfamilies of PDE4 enzymes (PDE4A-D) that hydrolyze the phosphodiester bond of cAMP, a key intracellular signaling molecule. Highlighting its physiological importance, *PDE4B* is classified by the Genome Aggregation Database (gnomAD) as extremely loss-of-function intolerant (Chen et al., 2024).

**Figure 1.**
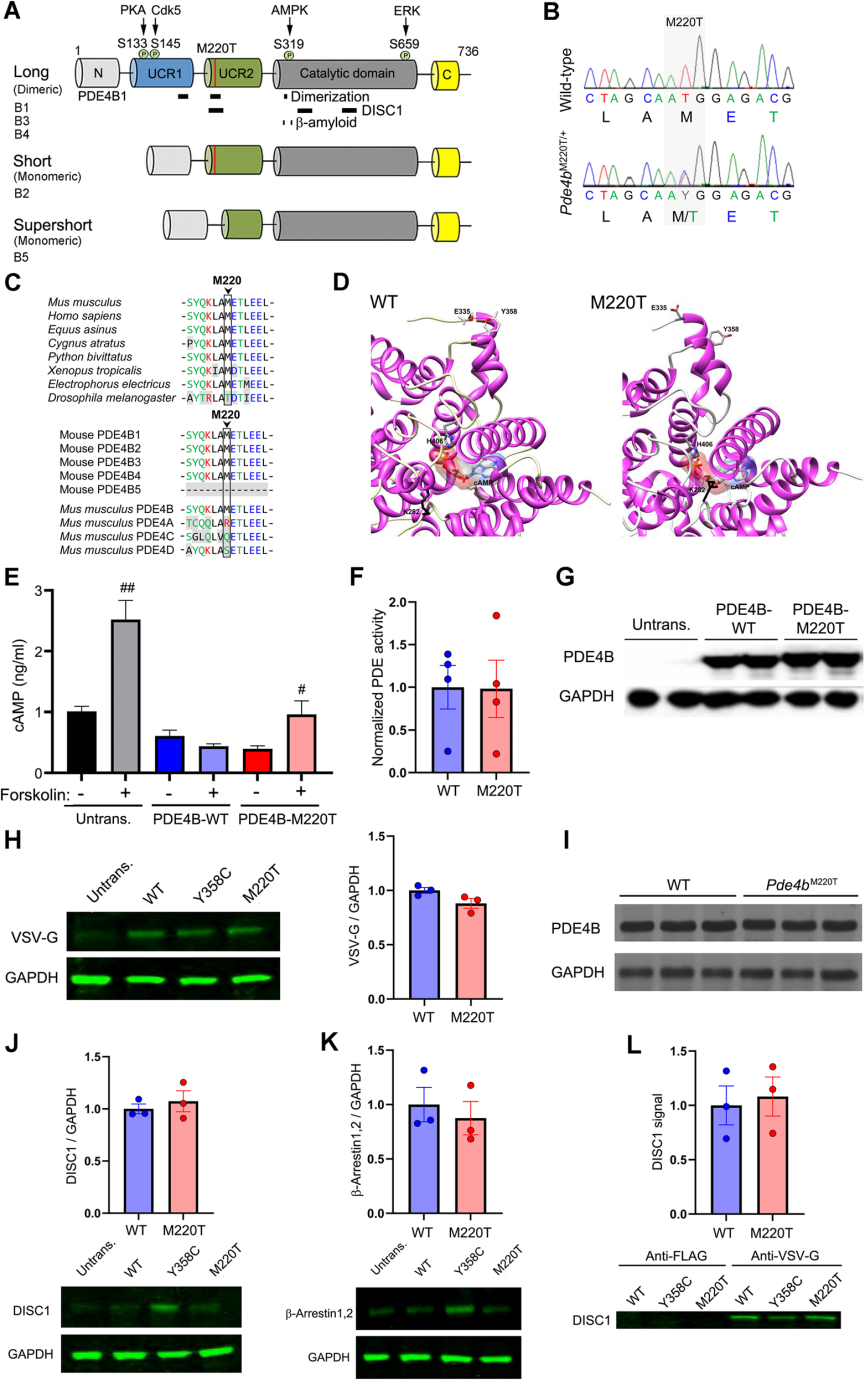
Functional consequences of PDE4B^M220T^ variant. ***A***, Schematic diagram depicting domain structure and functions of PDE4B isoforms. The serine residues phosphorylated by PKA (S133; activation), cyclin-dependent kinase 5 (Cdk5; S145; activation), AMP-activated protein kinase (AMPK; S319; activation), and extracellular signal-related kinase (ERK; S659; inhibition) are shown (Baillie et al., 2000; MacKenzie et al., 2002; Plattner et al., 2015; Johanns et al., 2016). Red vertical lines indicate M220T. Black horizontal lines indicate sites for PDE4B dimerization (residues 168–183, 217–235, and 314–316; Richter and Conti, 2002; Bolger et al., 2015; Cedervall et al., 2015), DISC1 binding (residues 212–245, 352–380, and 477–500; Murdoch et al., 2007), and β-amyloid binding (residues 312–313 and 327–328; Sin et al., 2024). ***B***, Sanger sequence chromatograms showing the c.659T>C transition in *Pde4b* exon 8, which is predicted to convert residue 220 in PDE4B1 from ATG methionine (M) to ACG threonine (T). ***C***, Top, Partial protein sequence alignment of mouse PDE4B1 and orthologs showing conservation in vertebrates of the M^220^ residue. Human PDE4B1 has a high degree of DNA (89%) and protein (97%) sequence homology with the mouse ortholog. Bottom, Partial protein sequence alignment of mouse PDE4B isoforms and paralogs showing that M^220^ is restricted to PDE4B1–4. PDE4B4 is not encoded by the human and orangutan genomes (Shepherd et al., 2003; Martin et al., 2023). Amino acid color code: black (nonpolar), green (uncharged polar), red (basic), blue (acidic). Residues disparate between mouse PDE4B1 and orthologs and paralogs have a gray background. ***D***, Left, Structural model of mouse PDE4B1-WT. Right, Structural model of mouse PDE4B1-M220T variant showing cAMP binding pocket conformation altered by a β conformational bend around K282 (black). The conformation of the cAMP binding pocket is altered by the introduction of a β conformational bend around K282 and an internal interaction between E335 and Y358 is abolished. ***E***, cAMP hydrolytic function of eGFP-tagged PDE4B1-WT transfected, eGFP-tagged PDE4B1-M220T transfected, and untransfected HEK-293T cells with (+) and without (−) forskolin treatment (10 μm; 30 min). The cAMP concentration is inversely related to cAMP hydrolysis. ***F***, cAMP hydrolytic function of VSV-G-tagged PDE4B1-WT and M220T constructs expressed in HEK-293 cells (unpaired *t* test: *t *= 0.04; *p *= 0.97). Mean of four experiments. ***G***, Unaltered PDE4B1 protein expression in eGFP-tagged PDE4B1-M220T versus PDE4B1-WT transfected HEK-293T cells. ***H***, Unaltered PDE4B1 protein expression in VSV-G-tagged PDE4B1-M220T versus PDE4B1-WT transfected HEK-293 cells (unpaired *t* test: *t *= 2.26; *p *= 0.08). Mean of three experiments. ***I***, Unaltered PDE4B protein expression in amygdala from *Pde4b*^M220T^ versus WT mice. ***J***, Unaltered endogenous DISC1 protein expression in VSV-G-tagged PDE4B1-M220T versus PDE4B1-WT transfected HEK-293 cells (unpaired *t* test: *t *= −0.66; *p *= 0.54). ***K***, Unaltered endogenous β-arrestin-1/2 protein expression in VSV-G-tagged PDE4B1-M220T versus PDE4B1-WT transfected HEK-293 cells (unpaired *t* test: *t *= 0.57; *p *= 0.6). ***L***, Unaltered PDE4B1-M220T binding to DISC1 demonstrated by coimmunoprecipitation of endogenous DISC1 in HEK-293 cells expressing VSV-G-tagged PDE4B1-M220T or PDE4B1-WT constructs (unpaired *t* test: *t *= −0.32; *p *= 0.76). VSV-G-tagged PDE4B1-Y358C was included as a positive control for decreased DISC1 binding (McGirr et al., 2016). Mean of three experiments. Data are plotted as mean ± SEM. ^#^*p *< 0.05; ^##^*p *< 0.01 versus untreated within each genotype. PDE, phosphodiesterase; Untrans., untransfected.

PDE4B is expressed as multiple isoforms (PDE4B1–5) via mRNA splicing, and each isoform contains a highly conserved C-terminal catalytic domain and either one or two N-terminal regulatory domains termed Upstream Conserved Region 1 and 2 (UCR1 and 2). The long forms PDE4B1, PDE4B3, and PDE4B4 contain both UCR1 and UCR2, the short form PDE4B2 lacks UCR1, and the supershort form PDE4B5 additionally lacks part of UCR2 (Fig. 1*A*; Cedervall et al., 2015). All five isoforms are present in adult mammalian brain tissue (Bunnage et al., 2015), and single-cell RNA sequencing has detected PDE4B mRNA in nearly all subclasses of GABAergic inhibitory neurons and glutamatergic excitatory neurons and in some types of glial cell (Armstrong et al., 2024). The lead SNPs for stress-related disorders (Meier et al., 2019; Nievergelt et al., 2024) and addiction risk (Hatoum et al., 2023), and the site of DNA methylation (cg14227435) that correlates with *PDE4B* expression and re-experiencing symptom severity in PTSD patients (Hori et al., 2024), are all located in *PDE4B* intron 3, thus implicating the long forms over the short forms.

To examine the effect of PDE4B variation on phenotypes with translational relevance to psychiatric disorders, we tested mice that replicate human missense variant M220T (rs775201287; 1-66332532-T-C) located in UCR2. Herein, we report that homozygous *Pde4b*^M220T^ mice exhibit increased PTSD-like behavior in response to trauma, as well as cortical hypoactivity and hyperactivity of the hippocampus and amygdala. Our findings provide substantive biological evidence linking PDE4B variation to PTSD-relevant phenotypes and thus highlight how genetic variation of PDE4B may contribute to PTSD risk.

## Materials and Methods

### Ethical approvals

Mouse experiments were approved by The Centre for Phenogenomics (TCP) Animal Care Committee and were conducted in compliance with the Ontario Animals for Research Act 1971 and Canadian Council on Animal Care guidelines.

### Generation of *Pde4b^M220T^* mutant

Genomic DNA from 7,502 F_1_ progeny of ENU-mutagenized C57BL/6JJcl males and untreated DBA/2JJcl females in the RIKEN BioResource Center frozen sperm archive was screened for mutations in *Pde4b* exon 8 (113 bp), as described previously (Sakuraba et al., 2005). In a single mouse (*Pde4b^Rgsc02383^*), we detected transition c.659T>C.

### Mice

Heterozygous N_2_ backcross progeny of the founder *Pde4b*^M220T^ heterozygous (DBA/2JJcl × C57BL/6JJcl) F_1_ male and wild-type (WT) C57BL/6JJcl females were backcrossed to C57BL/6JJcl (CLEA, Tokyo, Japan) for at least 10 generations before heterozygotes were intercrossed to generate homozygous mutant (*Pde4b*^M220T^) and WT littermates for phenotypic testing. Mice were genotyped for the M220T variant by the presence of a *Bsr*DI (R0574, New England Biolabs) restriction site. At 3 weeks of age, pups of mixed genotype were weaned and housed in groups of three to five same-sex littermates under controlled temperature (21 ± 1°C), lighting (lights on: 7 A.M.–7 P.M.), and humidity (50–60%). The C57BL/6JJcl congenic *Pde4b*^M220T^ (*Pde4b^Rgsc02383^*) line is cryopreserved at TCP, Toronto, Canada.

### Structural modeling

The mouse PDE4B1-WT protein and the M220T variant were modeled using the Phyre2 Protein Fold Recognition Server (Kelley et al., 2015).

### Mammalian expression constructs

M220T was introduced into ampicillin-resistant constructs pEE7.hCMV>VSV-G-hPDE4B1 (provided courtesy of Kirsty Millar, University of Edinburgh) and pRP[Exp]-EGFP-EF1A>hPDE4B1 (VectorBuilder) using a QuikChange II Site-Directed Mutagenesis Kit (Agilent). The M220T mutation was confirmed by DNA sequencing using a BigDye Terminator v3.1 Cycle Sequencing Kit (Applied Biosystems).

### Phosphodiesterase activity

To measure the cAMP hydrolytic activity of eGFP-tagged PDE4B1-M220T under basal conditions, HEK-293T cells were transfected with eGFP-tagged PDE4B1 constructs. At 48 h post-transfection, some HEK-293T transfectants were exposed to 10 µM forskolin (Enzo Life Sciences) for 30 min before all cells were lysed on ice. The intracellular cAMP concentration in the lysates was measured using a cAMP ELISA Kit (E-EL-0056, Elabscience Biotechnology). The phosphodiesterase activity of lysates from VSV-G-tagged PDE4B1 transfectants was determined using a two-step radioassay procedure, as described previously (Marchmont and Houslay, 1980; McGirr et al., 2016).

### Western blotting

Transfected HEK-293 cell lysate Western blots were carried out using mouse monoclonal anti-VSV glycoprotein (V5507, Sigma-Aldrich), mouse monoclonal anti-PDE4B (TA503471, OriGene Technologies), rabbit polyclonal anti-β-Arrestin-1/2 (sc-28869, Santa Cruz Biotechnology), rabbit polyclonal antibody to human DISC1 residues 683–832 (courtesy of Tetsu Akiyama, University of Tokyo; Ogawa et al., 2005), and mouse monoclonal anti-GAPDH (ab8245, Abcam), as described previously (McGirr et al., 2016). Western blotting of amygdala tissue dissected from 16-week-old male *Pde4b*^M220T^ and WT littermates was carried out using mouse monoclonal anti-PDE4B (1:2,000; TA503471, OriGene Technologies) and mouse monoclonal anti-GAPDH (1:10,000; ab8245, Abcam), as described previously (McGirr et al., 2016).

### Coimmunoprecipitation

To measure the effect of M220T on the binding of VSV-G-tagged PDE4B1 to endogenous DISC1, coimmunoprecipitation using mouse monoclonal anti-VSV glycoprotein–agarose antibody (A1970, Millipore) and a rabbit polyclonal antibody to human DISC1 (Ogawa et al., 2005) was conducted as described previously (McGirr et al., 2016). PDE4B1-Y358C was used as a positive control for decreased DISC1 binding (McGirr et al., 2016).

### Mouse behavioral testing

All behavioral experiments were conducted using naive male *Pde4b*^M220T^ and WT littermates at 10–16 weeks of age. Due to budgetary restrictions, female mice were not utilized. Mice were handled for 1 week prior to behavioral testing and transferred to the experimental room 30 min prior to the start of testing. Testing equipment was cleaned with 70% ethanol between each mouse. All experiments were conducted between 9 A.M. and 4 P.M.

#### Open field

Spontaneous locomotor activity was assessed in 1 h duration trials using a VersaMax Animal Activity Monitoring System (Omnitech Electronics), as described previously (Lipina et al., 2013).

#### Elevated plus maze

The elevated plus maze test was conducted using EthoVision XT video tracking software (Noldus), as described previously (Lipina et al., 2022).

#### Forced swim test

The forced swim test (FST) was conducted using The Observer 5.0 software (Noldus), as described previously (Lipina et al., 2012).

#### Three-chamber social approach test

The social approach test was conducted using a three-chambered box (each chamber 40 × 20 cm) and The Observer 5.0 software (Noldus), as described previously (Lipina et al., 2012).

#### Prepulse inhibition of acoustic startle response

Acoustic startle response (ASR) testing was conducted using a Startle Reflex System (ENV-022s, Med Associates), as described previously (Lipina et al., 2013). Pulse-only trials consisted of a single white noise burst (110 dB, 40 ms). Prepulse + pulse trials consisted of a prepulse of noise (20 ms at 72, 78, 82, or 86 dB) followed 100 ms after prepulse onset by a startling pulse (110 dB, 40 ms). No-stimulus trials consisted of background noise only (65 dB).

#### Morris water maze

Morris water maze (MWM) testing was conducted using 120-cm-diameter cylindrical tank filled with opaque water (40 cm depth; 24 ± 1°C), as described previously (Lipina et al., 2022). Video output from a camera above the pool center was analyzed using HVS Water 2020 software (HVS Image). Mice were given four training trials for 1 d to a visible platform at the center of the SE (target) quadrant, followed by 20 training trials (four per day) to a now submerged platform (1 cm below water surface) in the SE quadrant. A probe trial with the platform removed from the pool was given 18 h after the last training trial. The maximum duration of each trial was 60 s.

#### Object location test

The object location test (OLT) was conducted in a transparent acrylic arena (41 × 41 × 31 cm), using The Observer 5.0 software (Noldus), as described previously (Lipina et al., 2013). In training periods lasting for 5 or 15 min, a mouse was placed at the center of the arena and left to explore four identical plastic objects (inverted 100 ml beakers) placed at specific locations near each corner (5 cm from walls).

#### Y-maze

Y-maze testing was conducted using an apparatus consisting of three arms (40 × 8 × 15 cm; A, B, and C) made of gray opaque polyvinyl plastic placed at 120° from each other, as described previously (Lipina et al., 2013). The percentage of alternations was defined according to the following equation: % alternation = [(number of alternations) / (total arm entries − 2)] * 100.

#### Puzzle box

The puzzle box test was conducted using an apparatus consisting of a white acrylic arena divided by a removable barrier into two compartments: a brightly lit start zone (58 × 28 cm) and a smaller covered goal zone (15 × 28 cm), as described previously (Lipina et al., 2013).

#### Fear conditioning

Fear conditioning was conducted using a test chamber (25 × 30 × 25 cm; Med Associates) connected to a personal computer running FreezeFrame software (Actimetrics) that administered two CS–US pairings—auditory tone (3.6 kHz, 75 dB, 30 s) conditioned stimulus (CS) followed by footshock (0.75 mA, 2 s) unconditioned stimulus (US)—delivered 60 s apart, as described previously (Lipina et al., 2013). In the contextual memory test, 24 h after conditioning, the mouse was returned to the chamber and monitored for 8 min. In the cued fear memory test, 48 h after conditioning, the mouse was placed in an altered chamber (novel odor, lighting, background noise, floor, shape, visual cues) and allowed to explore for 3 min before the auditory tone was presented continuously for 8 min.

To model a traumatic event, mice were subject to fear conditioning with 10 CS–US pairings (trauma), 60 s apart, of an auditory tone (3.6 kHz, 75 dB, 30 s) CS immediately followed by a footshock (1 mA, 2 s) US, as described previously with slight modifications (Bliss et al., 2010). At 6, 24 and 48 h after conditioning, cued fear memory was assessed in an altered chamber, as described above. Separate cohorts of mice were assessed for cued fear memory in response to the CS at 3, 15 or 30 d after conditioning.

#### Ultrasonic vocalization

Ultrasonic vocalization (USV) during the last 3 min of cued fear memory testing was recorded using an UltraSoundGate ultrasonic microphone and RECORDER software (Avisoft Bioacoustics). USVs emitted in the 22–35 kHz range, corresponding to fear-related calls (Wöhr and Schwarting, 2013), were analyzed using SASLab Pro software (Avisoft Bioacoustics), as described previously (Mun et al., 2015a).

#### Hotplate test of pain sensitivity

Pain sensitivity was measured using a Hot Plate Analgesia Meter (Columbus Instruments) set at a constant temperature of 52.5 ± 0.5°C, as described previously (Wilkerson et al., 2018).

### Corticosterone measurement

Plasma levels of corticosterone in blood samples collected from naive (nontrauma exposed) mice and trauma-exposed mice at 24 h and 30 d post-trauma, 30 min after re-exposure to the auditory tone (CS), were measured using a Corticosterone ELISA Kit (Cayman Chemical) according to manufacturer's instructions. Each sample was run in triplicate.

### Hippocampal BDNF

BDNF levels in the hippocampi dissected from the same mice killed for measurement of plasma corticosterone levels, at baseline (nontrauma exposed) and at 24 h and 30 d post-trauma, 30 min after re-exposure to the auditory tone (CS), were measured using a Mouse BDNF Sandwich ELISA kit (LS-F2404, LifeSpan BioSciences), as described previously (Xian et al., 2019).

### Immunohistochemistry staining for c-Fos protein

The number of c-Fos-positive cells in brain sections (50 µm thick) from mice, 90 min after trauma, was measured using rabbit polyclonal anti-c-Fos (1:500; sc-52, Santa Cruz Biotechnology) and Alexa Fluor 488-conjugated goat polyclonal anti-rabbit IgG (1:1,000; ab150077, Abcam), as described previously (Mun et al., 2015b). For quantitative analysis, 6–7 mice per genotype and 4–5 sections per mouse were used.

### Statistical analyses

Statistical analyses were performed using Statistica 14.0 (TIBCO Software). Data were tested for normality using the Shapiro–Wilk test and for homoscedasticity using Levene's test. Data passing normality and homoscedasticity assumptions were analyzed using one-way, two-way, or three-way analysis of variance (ANOVA) with repeated measures (RM), as necessary, followed by Tukey’s post hoc tests with statistical significance set at *p *< 0.05. Pearson's correlation coefficients (*r*) were calculated to assess relationships between the percentage of freezing in cued fear memory testing and the amplitude of fear-related calls or c-Fos staining and between c-Fos staining in different brain regions. The exact number of samples included is given in each figure legend. Each sample corresponds to an individual dot in the graphs, which were prepared using Prism 8 software (GraphPad Software). Experimenters were blinded to genotype during behavioral testing.

## Results

### Biochemical consequences of *PDE4B^M220T^* variant

The c.659T>C transition in mutant transcript *Pde4b^Rgsc02383^* results in the nonpolar, hydrophobic methionine (M) at position 220 (PDE4B1 numbering) being replaced by a polar, hydrophilic threonine (T) in PDE4B1–4 (Fig. 1*A*–*C*). Structural modeling of the mouse PDE4B1-M220T variant predicted subtle changes compared with PDE4B1 wild-type (WT), but not to the extent of disrupting the normal orientation and binding of cAMP (Fig. 1*D*).

In HEK-293 cells expressing eGFP- or VSV-G-tagged constructs, the enzymatic activity of the PDE4B1-M220T variant was not significantly different compared with PDE4B1-WT under basal conditions (Fig. 1*E*,*F*). When challenged with the adenylyl cyclase activator forskolin (10 µm; 30 min), which induces phospho-activation of PDE4 by protein kinase A (PKA; Plattner et al., 2015), untransfected cells showed an expected increase in intracellular cAMP concentration compared with basal conditions. This forskolin-elicited increase was prevented by expression of eGFP-tagged PDE4B1-WT but was only partially attenuated by expression of eGFP-tagged PDE4B1-M220T (Fig. 1*E*), suggesting that the PDE4B1-M220T variant was less able to respond to a rapid rise in intracellular cAMP.

Western blotting revealed that in vitro PDE4B^M220T^ protein expression was unaltered in PDE4B1-transfected cells (Fig. 1*G*,*H*), a finding paralleled in vivo in amygdala tissue from *Pde4b*^M220T^ mice (Fig. 1*I*). PDE4B1-M220T-transfected cells also showed unaltered levels of endogenous DISC1 and β-arrestin-1/2 (Fig. 1*J*,*K*), known binding partners of PDE4B (Perry et al., 2002; Millar et al., 2005; Cheung et al., 2007; Murdoch et al., 2007). M^220^ is located within the DISC1 binding site in UCR2, but coimmunoprecipitation of endogenous DISC1 was unaltered in PDE4B1-M220T-transfected cells (Fig. 1*L*).

### *Pde4b*^M220T^ mice exhibit heightened reactivity to novel environments

**Figure 2.**
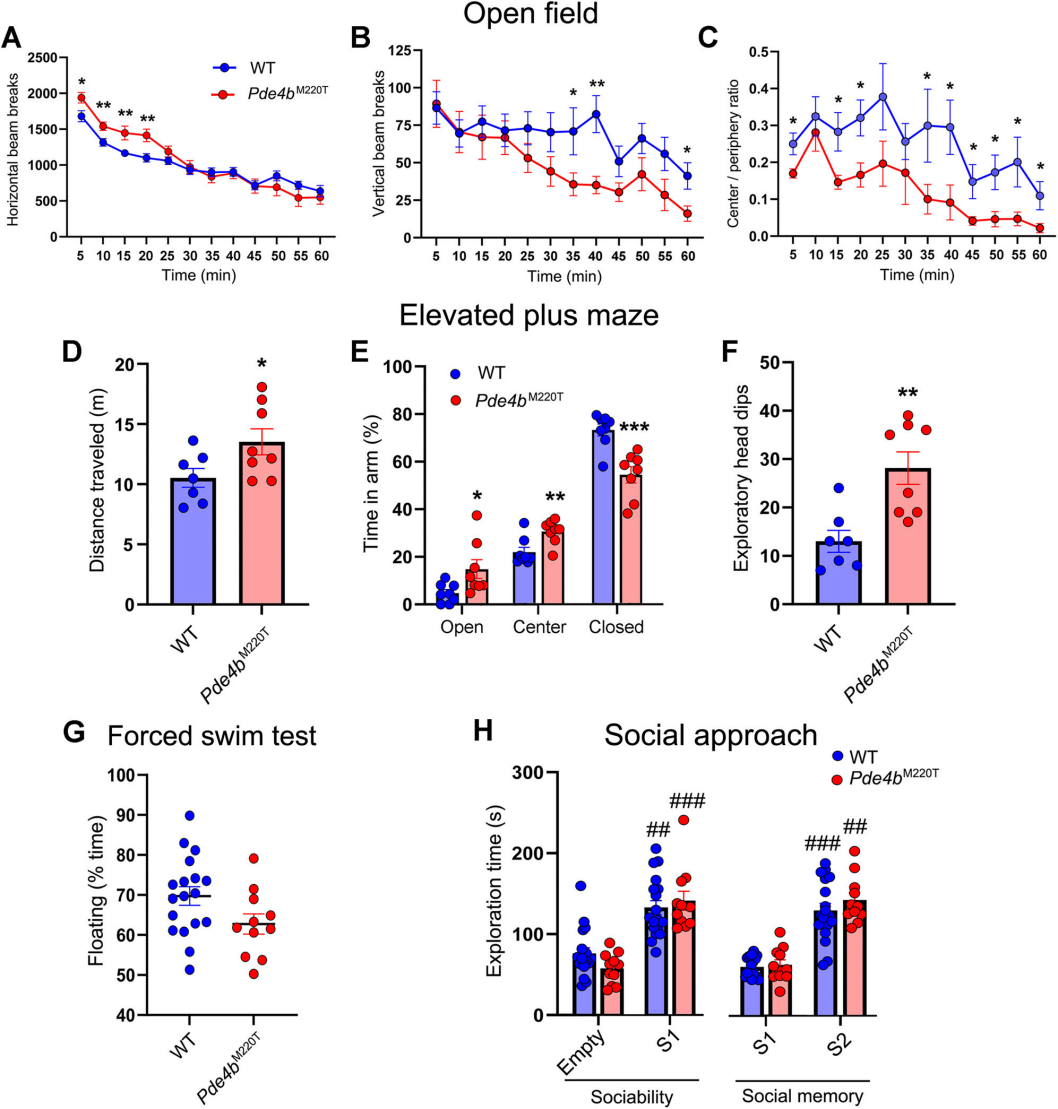
Behavior of *Pde4b*^M220T^ mice in open field, EPM, forced swim, and social approach tests. ***A***, OF: locomotor activity quantified as number of horizontal beam breaks in 5 min intervals. *Pde4b*^M220T^ mice were more active during the first 20 min (RM ANOVA, time: *F*_(11,297) _= 82.2, *p *< 0.0001; genotype × time interaction: *F*_(11,297) _= 4.7, *p *< 0.0001). ***B***, OF: rearing quantified as number of vertical beam breaks. *Pde4b*^M220T^ mice reared less than WT mice at 30–40 min and 55–60 min (RM ANOVA, time: *F*_(11,297) _= 13.6, *p *< 0.0001; genotype × time interaction: *F*_(11,297) _= 2.9, *p *< 0.001). ***C***, OF: fraction of time in the center versus the periphery in 5 min intervals (RM ANOVA, genotype: *F*_(1,27) _= 4.4, *p *< 0.05; time: *F*_(11,297) _= 5.6; *p *< 0.0001). ***D***, EPM: distance traveled (m). ***E***, EPM: percentage of time in the open arms (*F*_(1,14) _= 5.7; *p *< 0.05), closed arms (*F*_(1,14) _= 19.8; *p *< 0.001), and central square (*F*_(1,14) _= 10.7; *p *< 0.01). ***F***, EPM: number of exploratory head dips. ***G***, FST, percentage of time spent floating. ***H***, Social approach test. Sociability: time (s) spent exploring an empty container versus a novel mouse (stranger 1; RM ANOVA, genotype: *F*_(1,27) _= 0.8, *p *> 0.05; stranger 1: *F*_(1,27) _= 17.7, *p *< 0.01). Social memory: time (s) spent exploring stranger 1 (previously explored mouse) versus a second novel mouse (stranger 2; RM ANOVA, genotype: *F*_(1,27) _= 2.5, *p *> 0.05; stranger 2: *F*_(1,27) _= 33.3, *p *< 0.001). Data are plotted as mean ± SEM. *p < 0.05; **p < 0.01; ***p < 0.001 versus WT. ^##^*p *< 0.01; ^###^*p *< 0.001 versus empty container or stranger 1 within each genotype. Empty, empty cylinder; S1, stranger 1; S2, stranger 2. Additional data are shown in Extended Data Figure 2-1 (EPM) and 2–2 (social approach).

Since GWAS data have associated *PDE4B* with PTSD (Gelernter et al., 2019; Meier et al., 2019; Nievergelt et al., 2024) and schizophrenia (Trubetskoy et al., 2022), which are often comorbid (Dallel et al., 2018), we subjected *Pde4b*^M220T^ mice to several behavioral tests that examine a range of evolutionary conserved cognitive and behavioral domains. To examine the behavioral response of *Pde4b*^M220T^ mice to a mildly stressful novel environment, we measured their locomotor activity in an open field (OF) over 1 h. *Pde4b*^M220T^ mice were more active during the first 20 min, but their locomotor activity was comparable with WT mice thereafter (Fig. 2*A*), suggesting that the heightened initial activity was in response to the novelty of the environment. Both genotypes showed a similar decline in locomotor activity over time, indicative of unaltered habituation. *Pde4b*^M220T^ mice exhibited less rearing at 30–40 min and 55–60 min than WT mice (Fig. 2*B*). *Pde4b*^M220T^ mice also spent a lower fraction of time at the center versus the periphery of the arena than WT mice (Fig. 2*C*), demonstrating greater thigmotaxis.

10.1523/JNEUROSCI.0137-24.2024.f2-1 Figure 2-1 Behavior of *Pde4b*^M220T^ and WT mice in the elevated plus maze. *Pde4b*^M220T^ mice made more entries to the open arms, closed arms and center, and more passages between the open arms, than WT mice. *p < 0.05, **p < 0.01, ***p < 0.001 versus WT. ANOVA, analysis of variance; N^o.^, number of; WT, wild-type. Download Figure 2-1, DOCX file.

To probe the response of *Pde4b*^M220T^ mice to novel environments further, we examined their behavior in an elevated plus maze (EPM), consisting of two wall-enclosed arms intersected by two open arms. Over the 5 min test, *Pde4b*^M220T^ mice ambulated further, spent a greater percentage of time in the open arms, and made more exploratory head dips (Fig. 2*D*–*F*) and passages between the open arms (Extended Data Fig. 2-1) than WT mice. *Pde4b*^M220T^ mice thus displayed exaggerated locomotor activity responses to two different novel environments, indicating heightened reactivity to environmental novelty. *Pde4b*^M220T^ mice did not display differences in the FST and the social approach test (Fig. 2*G*,*H*; Extended Data Fig. 2-2).

10.1523/JNEUROSCI.0137-24.2024.f2-2 Figure 2-2 Behavior of *Pde4b*^M220T^ and WT mice in the three-chamber social approach test. *Pde4b*^M220T^ mice did not display differences in habituation, sociability or social memory. N^o.^, number of; WT, wild-type. Download Figure 2-2, DOCX file.

### *Pde4b*^M220T^ mice exhibit increased startle reactivity and decreased prepulse inhibition

**Figure 3.**
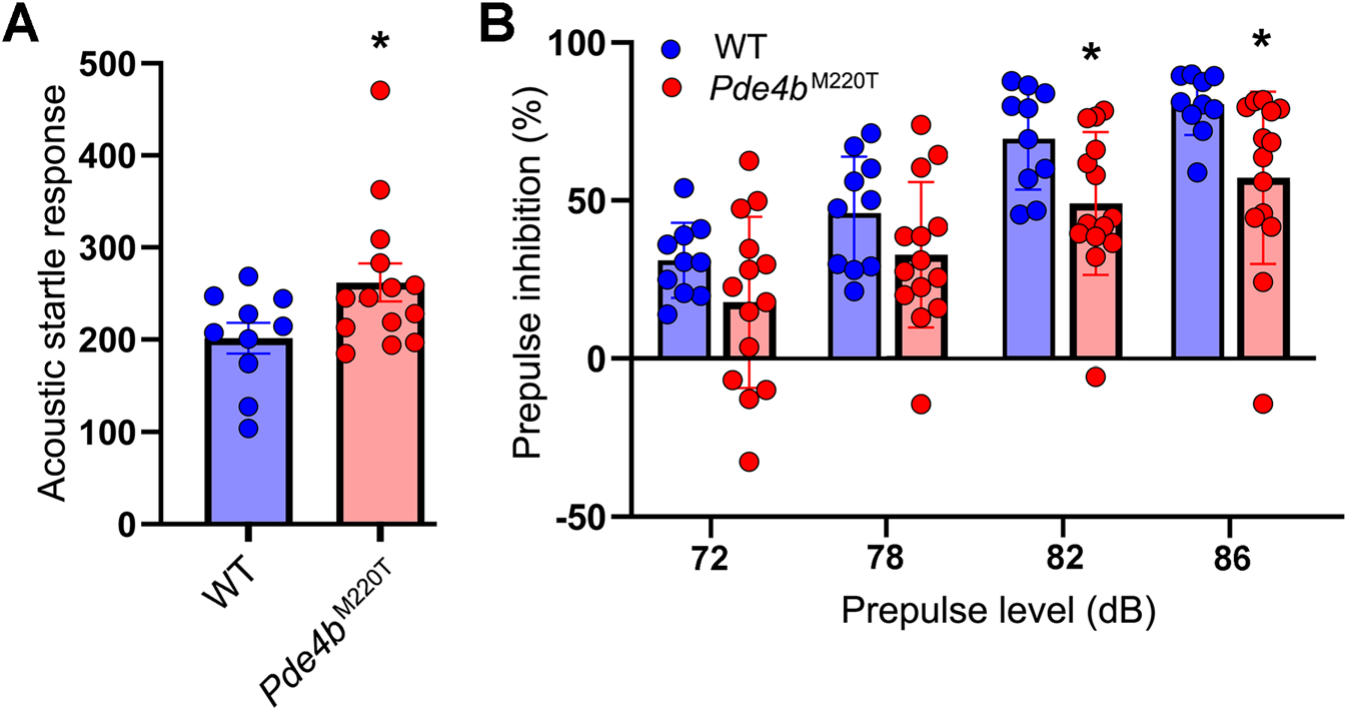
Acoustic startle response and prepulse inhibition in *Pde4b*^M220T^ mice. ***A***, Amplitude of acoustic startle response (ASR) to a startle stimulus (110 dB, 40 ms; RM ANOVA, genotype: *F*_(1,22) _= 4.6; *p *< 0.05). ***B***, Prepulse inhibition of the ASR expressed as the percent reduction in startle amplitude at prepulse levels of 72, 78, 82, and 86 dB (RM ANOVA, genotype: *F*_(1,22) _= 5.1, *p *< 0.05; prepulse: *F*_(3,66) _= 71.8, *p *< 0.0001). Data are plotted as mean ± SEM. *p < 0.05 versus WT. dB, decibels.

The acoustic startle reflex is a coordinated contraction of the skeletal musculature in response to a loud and unexpected noise. An exaggerated startle reflex is one of the clinical symptoms of PTSD (Jovanovic et al., 2009; American Psychiatric Association, 2013). When presented with a startling pulse of sound (110 dB, 40 ms), *Pde4b*^M220T^ mice displayed a greater acoustic startle response (ASR) than WT mice (Fig. 3*A*). Prepulse inhibition (PPI) refers to the amygdala-modulated attenuation of the ASR when a brief low-intensity prepulse shortly precedes the startle-eliciting pulse (Cano et al., 2021). When presented with prepulses of 82 and 86 dB before the 110 dB startle pulse, *Pde4b*^M220T^ mice displayed less PPI than WT mice, but PPI with prepulses of 72 and 78 dB was not significantly different between genotypes (Fig. 3*B*).

### *Pde4b*^M220T^ mice exhibit enhanced hippocampus-dependent spatial memory

**Figure 4.**
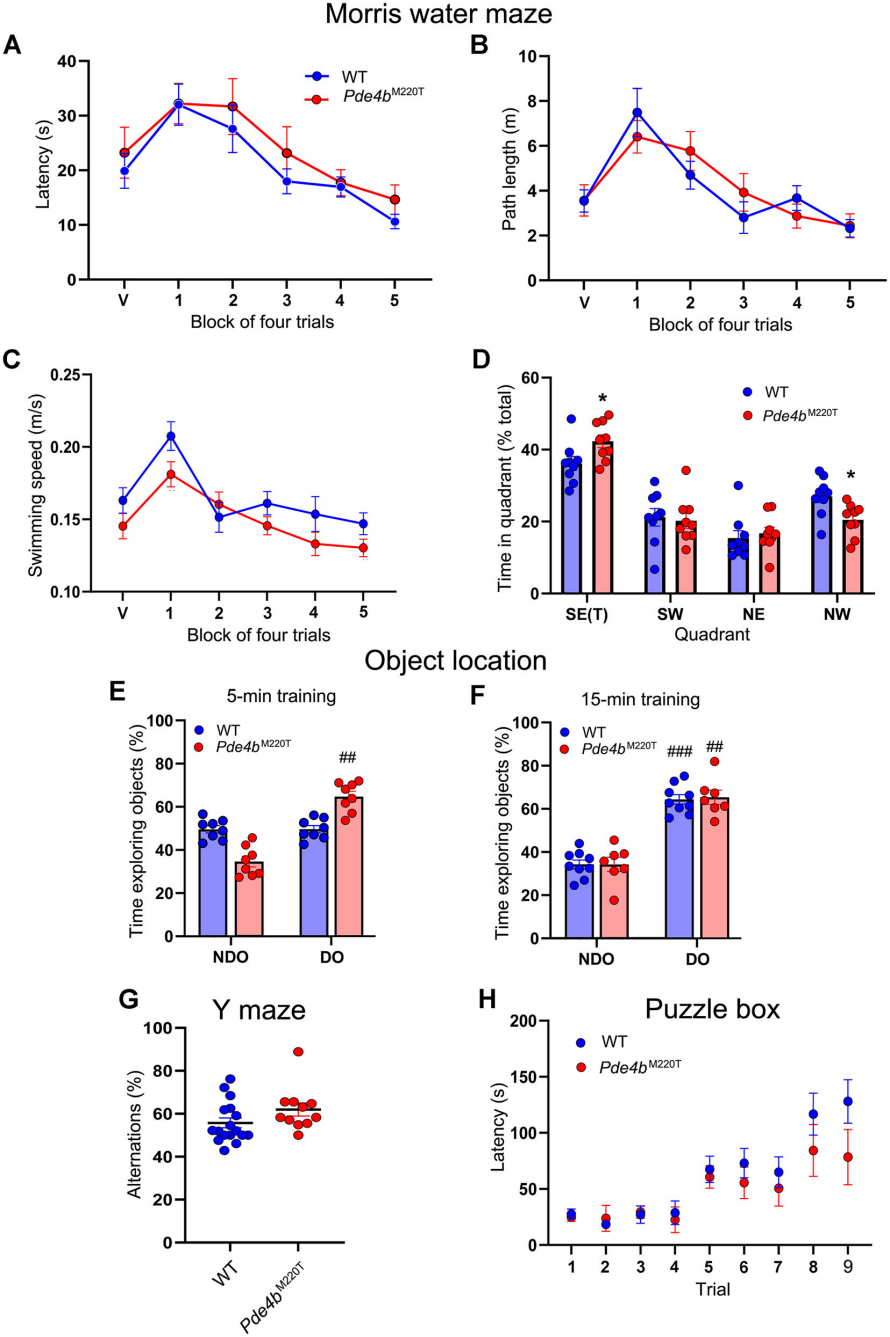
Hippocampus-dependent spatial memory in *Pde4b*^M220T^ mice. ***A***, MWM training trials: escape latency (s) at a block of four training trials with a visible platform (block V) and five blocks of four training trials with a submerged (hidden) platform in the southeast (SE) quadrant (blocks 1–5; RM ANOVA, genotype: *F*_(1,16) _= 1.89; *p *> 0.05; trial block: *F*_(5,80) _= 7.2, *p *< 0.001). ***B***, MWM training trials: path length (m) at a block of four training trials with a visible platform (block V) and five blocks of four training trials with a submerged (hidden) platform in the southeast (SE) quadrant (blocks 1–5). ***C***, MWM training trials: swimming speed (m/s) at each block of four training trials. ***D***, MWM probe trial: percentage of time spent in each quadrant of the pool after 20 training trials to the SE target (T) quadrant (RM ANOVA, quadrant: *F*_(3,48) _= 41.1, *p *< 0.0001; genotype × quadrant interaction: *F*_(3,48) _= 3.5; *p *< 0.05). *n *= 8–14 mice per genotype. ***E***, OLT (5 min training): percentage of time spent exploring nondisplaced (NDO) versus displaced (DO) objects after a 5 min training period (RM ANOVA, displaced object: *F*_(1,14) _= 6.2, *p *< 0.05; genotype × displaced object interaction: *F*_(1,14) _= 4.9, *p *< 0.01). ***F***, OLT (15 min training): percentage of time spent exploring nondisplaced (NDO) versus displaced (DO) objects after a 15 min training period (RM ANOVA, displaced object: *F*_(1,14) _= 12.9, *p *< 0.001). ***G***, Y-maze: percentage of alternation. ***H***, Puzzle box: latency (s) to enter the dark compartment of the puzzle box (goal). Data are plotted as mean ± SEM. *p < 0.05 versus WT. ^##^*p *< 0.01; ^###^*p *< 0.001 versus NDO within each genotype. T, target quadrant. Additional data are shown in Extended Data Figure 4-1 (OLT).

In the visible platform (nonspatial) version of the MWM, latency to reach a visible escape platform was not significantly different between genotypes (Fig. 4*A*). In the hippocampus-dependent hidden platform (spatial) version of the MWM, latency and path length to reach a submerged (hidden) escape platform and swimming speed over 5 d of training were not significantly different between genotypes (Fig. 4*A*–*C*), thus demonstrating unaltered spatial learning in *Pde4b*^M220T^ mice. During a probe trial with the platform removed, 18 h after the last training trial, the number of crossings of the former platform location was not significantly different between genotypes (*Pde4b*^M220T^: 8.2 ± 0.6; WT: 8.3 ± 0.8). However, *Pde4b*^M220T^ mice spent more time than WT mice in the target quadrant (Fig. 4*D*), demonstrating a more focused search of the pool.

10.1523/JNEUROSCI.0137-24.2024.f4-1 Figure 4-1 Behavior of *Pde4b*^M220T^ and WT mice in the object location test. Times spent freezing and exploring all objects were not significantly different between genotypes. WT, wild-type. Download Figure 4-1, DOCX file.

In the OLT (Assini et al., 2009), only *Pde4b*^M220T^ mice demonstrated a preference for displaced objects after a 5 min training period (Fig. 4*E*), but preference for displaced objects was not significantly different between genotypes after the training period was lengthened to 15 min (Fig. 4*F*; Extended Data Fig. 4-1). *Pde4b*^M220T^ mice did not display differences in the Y-maze test (Sarnyai et al., 2000) and the puzzle box test (Ben Abdallah et al., 2011; Fig. 4*G*,*H*). *Pde4b*^M220T^ mice thus demonstrated enhanced hippocampus-dependent spatial memory, without alterations in nonhippocampal working memory and executive functioning.

### *Pde4b*^M220T^ mice exhibit altered cued fear conditioning

**Figure 5.**
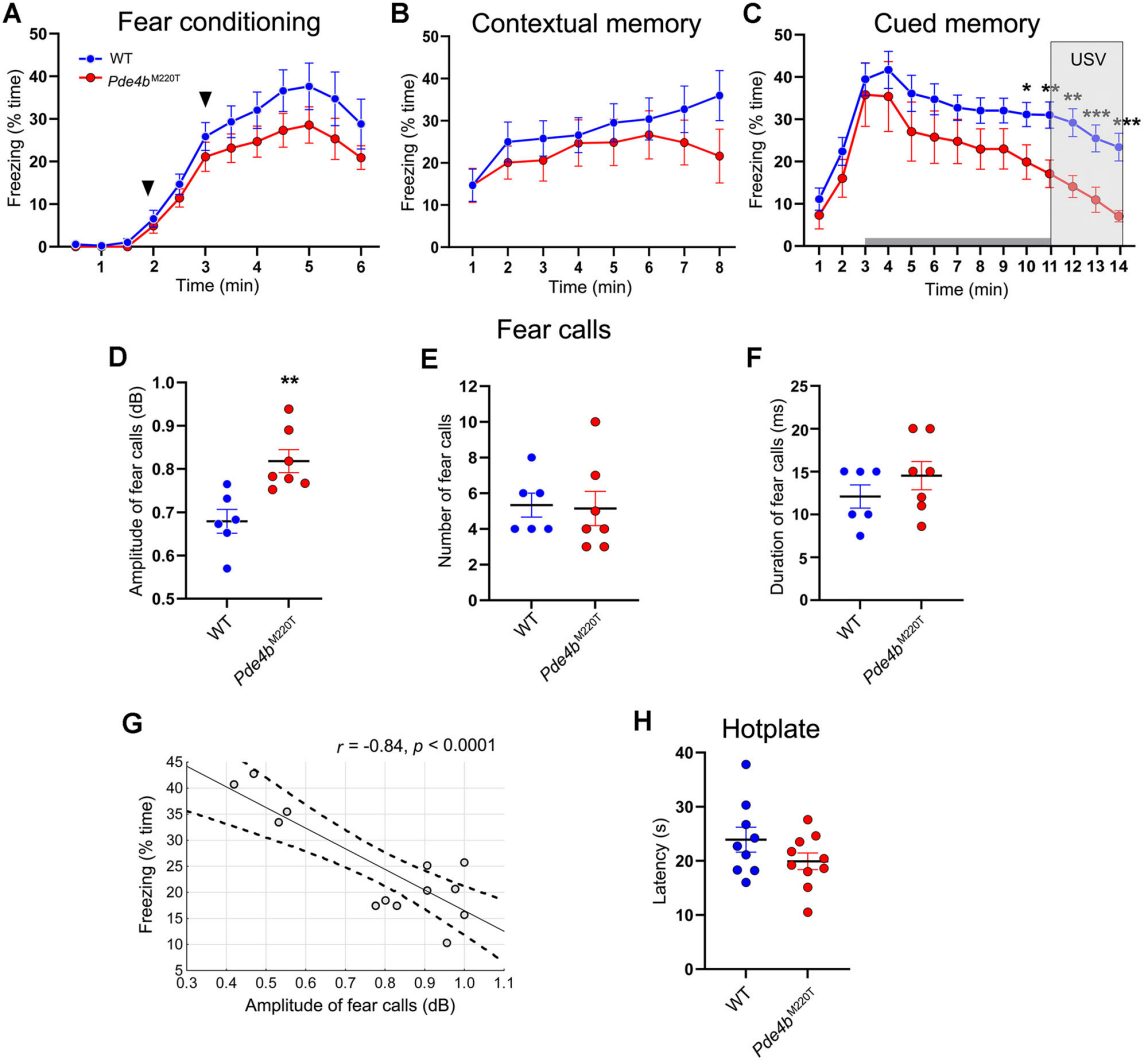
Contextual and cued fear conditioning in *Pde4b*^M220T^ mice. ***A***, Conditioning: percentage of time spent freezing during conditioning, when mice received two CS–US (tone–shock) pairings (arrowheads), in 30 s intervals (RM ANOVA, genotype: *F*_(1,13) _= 0.4, *p *> 0.05; time: *F*_(10,130) _= 6.7, *p *< 0.001; genotype × time interaction: *F*_(10,130) _= 0.5, *p *> 0.05). ***B***, Contextual memory test, 24 h after conditioning: percentage of time spent freezing in 1 min intervals (RM ANOVA, genotype: *F*_(1,17) _= 0.4, *p *> 0.05; time: *F*_(7,119) _= 8.0, *p *< 0.001; genotype × time interaction: *F*_(7,119) _= 1.4, *p *> 0.05). ***C***, Cued fear memory test, 48 h after conditioning: percentage of time spent freezing in 1 min intervals, with the 8 min auditory tone (gray box) presented at 3–11 min (RM ANOVA, genotype: *F*_(1,19) _= 0.7, *p *> 0.05; time: *F*_(13,247) _= 23.9, *p *< 0.001; genotype × time interaction: *F*_(13,247) _= 7.6, *p *< 0.05). ***D***, Amplitude of fear-related calls (USV at 22–30 kHz; dB) during the last 3 min of the cued fear memory test. ***E***, Number of fear-related calls. ***F***, Duration (ms) of fear-related calls. Data are plotted as mean ± SEM. USV, ultrasonic vocalization. ***G***, Correlation between amplitude of fear-related calls and percentage of freezing in the cued fear memory test. *n *= 6–7 mice per genotype. ***H***, Hotplate: Latency (s) to withdraw paw from hotplate (52.5 ± 0.5°C). Data are plotted as mean ± SEM. *p < 0.05; **p < 0.01; ***p < 0.001 versus WT. dB, decibels; USV, ultrasonic vocalization.

Mammals exhibit a repertoire of species-specific involuntary active (fight-or-flight) and passive defense responses to threats. For mice, freezing (absence of movement) is the dominant postencounter defensive response (Fanselow, 1994). In fear conditioning testing, mice demonstrate that they associate an environment (context) or an auditory tone (cue) with an aversive stimulus (electric footshock) by freezing, which is used as an index of fear memory. During conditioning, when mice received two tone–shock pairings (0.75 mA), the percentage of time spent freezing was not significantly different between genotypes (Fig. 5*A*).

In the contextual memory test, 24 h after conditioning, the percentage of freezing was not significantly different between genotypes (Fig. 5*B*), demonstrating unaltered contextual fear conditioning in *Pde4b*^M220T^ mice. In the cued fear memory test, 48 h after conditioning, *Pde4b*^M220T^ mice froze less than WT mice during the last 2 min of the auditory tone and the closing 3 min (Fig. 5*C*). *Pde4b*^M220T^ mice also issued louder USVs at frequencies of 22–30 kHz—termed fear-related calls (Wöhr and Schwarting, 2013)—than WT mice during the closing 3 min (Fig. 5*D*). However, the number and duration of fear-related calls was not significantly different between genotypes (Fig. 5*E*,*F*). Correlation analysis revealed a significant negative association between the amplitude of fear-related calls and the percentage of freezing (*r *= −0.84; *p *< 0.0001; Fig. 5*G*). Following conditioning with two tone–shock pairings, the defense response of *Pde4b*^M220T^ mice to the auditory tone was thus more active (flight/escape) and less passive (freezing) than WT mice. In a hotplate test, *Pde4b*^M220T^ mice showed unaltered pain sensitivity (Fig. 5*H*).

### *Pde4b*^M220T^ mice exhibit an altered response to trauma

**Figure 6.**
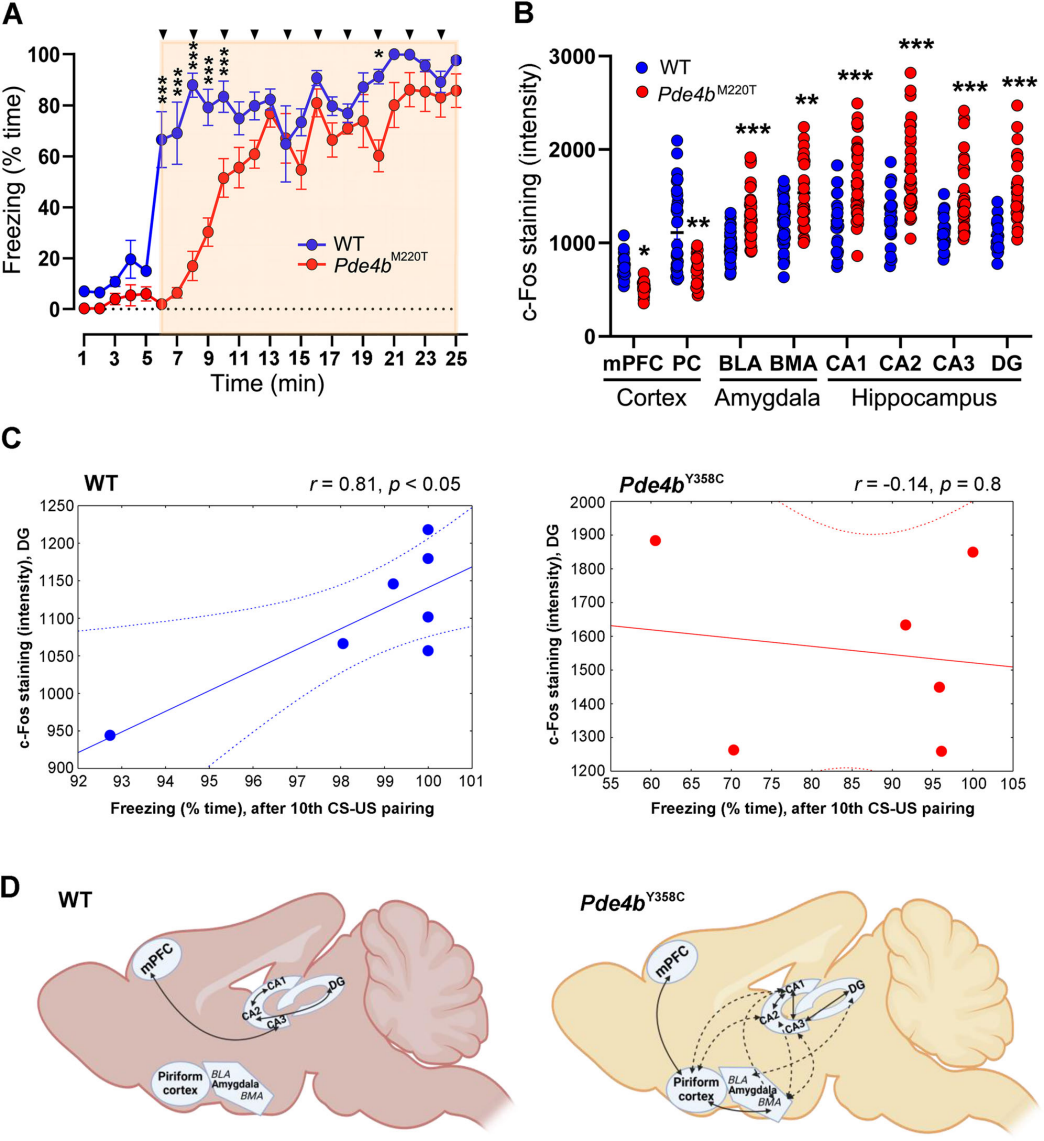
Brain neuronal activity in trauma-exposed *Pde4b*^M220T^ mice. ***A***, Exposure to trauma: percentage of time spent freezing during conditioning, before and during (shaded) the administration of ten CS–US (tone–shock) pairings (arrowheads) at 1 min intervals (traumatic event; RM ANOVA, genotype: *F*_(1,10) _= 20.2, *p *< 0.001; time: *F*_(24,240) _= 68.1; *p *< 0.0001; genotype × time interaction: *F*_(24,240) _= 7.5, *p *< 0.0001). ***B***, Genotypic difference in c-Fos staining (intensity of fluorescence) in the cortex (mPFC: *F*_(1,52) _= 18.5, *p *< 0.0001; PC: *F*_(1,80) _= 20.1, *p *< 0.01), amygdala (BLA: *F*_(1,76) _= 15.3, *p *< 0.0001; BMA: *F*_(1,69) _= 9.2, *p *< 0.01), and hippocampal formation (CA1: *F*_(1,58) _= 16.9, *p *< 0.0001; CA2: *F*_(1,53) _= 21.7, *p *< 0.0001; CA3: *F*_(1,56) _= 19.6, *p *< 0.0001; DG: *F*_(1,45) _= 33.7, *p *< 0.0001) in trauma-exposed mice at 90 min post-trauma. The area of each brain region examined is given in Extended Data Figure 6-1. ***C***, Correlation between c-Fos staining in the DG and the percentage of freezing during 1 min after the 10th tone–shock pairing. *n *= 6–7 mice per genotype. ***D***, Schematic diagrams depicting correlations between c-Fos staining in different brain regions in *Pde4b*^M220T^ and WT mice at 90 min post-trauma. Solid lines, positive correlations; broken lines, negative correlations. Created with BioRender.com. Pearson's correlation coefficients (*r*) are given in Extended Data Figure 6-2. Data are plotted as mean ± SEM. *p < 0.05; **p < 0.01; ***p < 0.001 versus WT. BLA, basolateral amygdala; BMA, basomedial amygdala; CA1, cornu ammonis 1; CA2, cornu ammonis 2; CA3, cornu ammonis 3; DG, dentate gyrus; mPFC, medial prefrontal cortex; PC, piriform cortex.

To assess whether *Pde4b*^M220T^ mice exposed to a greater threat would switch from an active to a passive defensive mode (Fanselow, 1994), a separate cohort of mice was conditioned with a more aversive stimulus of 10 tone–shock pairings and a more intense footshock (1.0 mA), modeling a traumatic event. By definition, an environmental stressor (traumatic event exposure) is required for the expression of PTSD. During conditioning, *Pde4b*^M220T^ mice froze less than WT mice during the first three tone–shock pairings (Fig. 6*A*).

10.1523/JNEUROSCI.0137-24.2024.f6-1 Figure 6-1 Area of each brain region examined for c-Fos-positive neurons in *Pde4b*^M220T^ and WT mice at 90  min post-trauma. Brain regions in the cortex, amygdala and hippocampal formation were not significantly different in area (number of pixels) between genotypes. WT, wild-type. Download Figure 6-1, DOCX file.

10.1523/JNEUROSCI.0137-24.2024.f6-2 Figure 6-2 Pearson’s correlation coefficients (*r*) between c-Fos staining in different brain regions in *Pde4b*^M220T^ and WT mice at 90  min post-trauma. In WT mice, the c-Fos staining showed significant positive correlations between subfields of the hippocampal formation (CA1–CA2 and CA2–DG) and between the CA3 and mPFC. In *Pde4b*^M220T^ mice, there was a more complex pattern of correlations between brain regions, with only the positive CA1–CA2 correlation in common with WT mice. Bold text, significant correlation; red text, common between WT and *Pde4b*^M220T^ mice; *n *= 4-5 sections/mouse. Download Figure 6-2, DOCX file.

**Figure 7.**
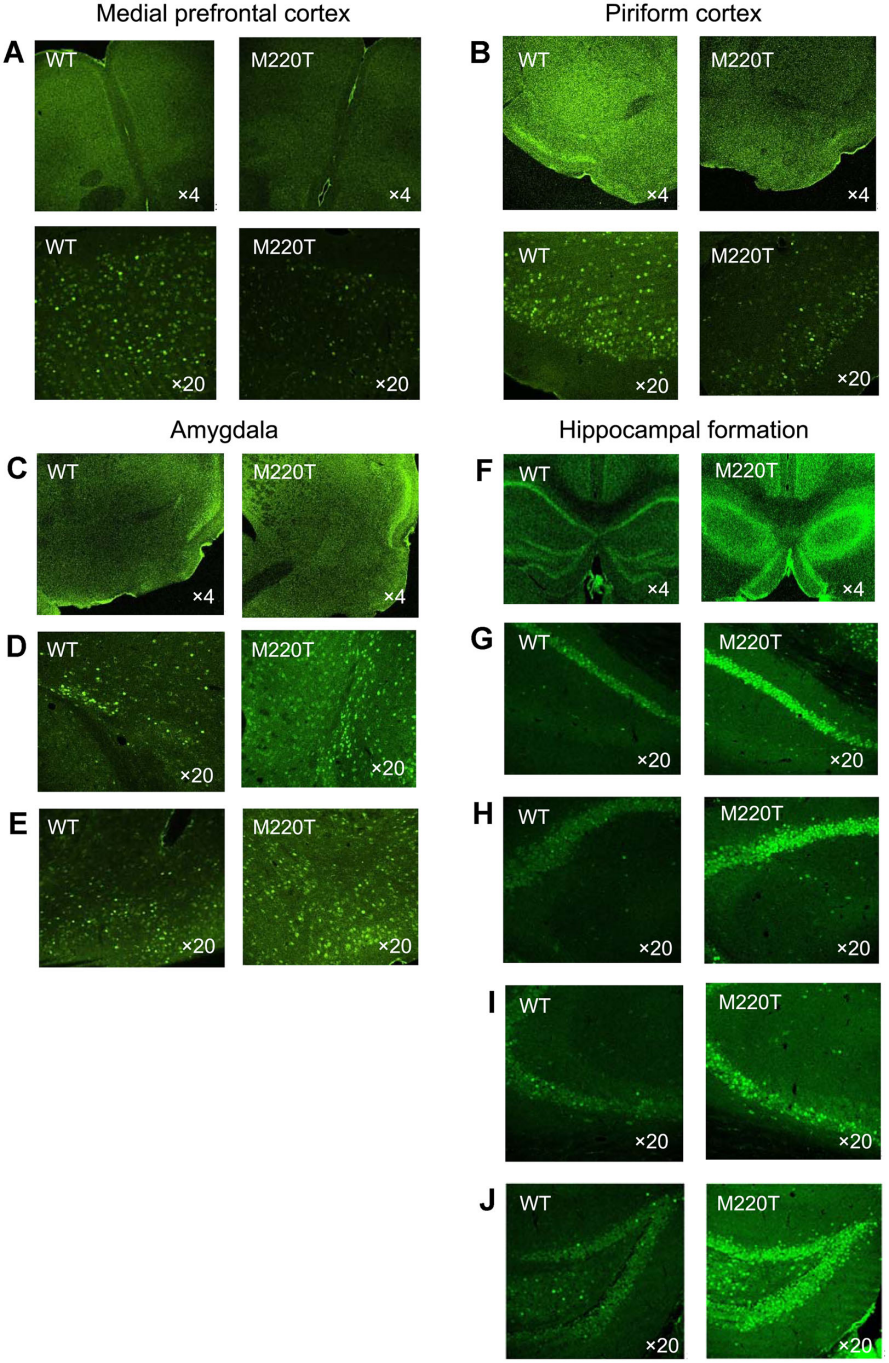
Representative images of c-Fos-positive neurons in the cortex, amygdala, and hippocampal formation in trauma-exposed mice at 90 min post-trauma. ***A***, Medial prefrontal cortex. ***B***, Piriform cortex. ***C***, Amygdala. ***D***, Basolateral amygdala. ***E***, Basomedial amygdala. ***F***, Hippocampal formation. ***G***, CA1. ***H***, CA2. ***I***, CA3. ***J***, Dentate gyrus. × 4 and ×20 magnification.

At 90 min post-trauma, the neuronal activity in brains from a subset of trauma-exposed mice was assessed by quantifying the neuronal activity-dependent expression of the transcription factor c-Fos. Trauma-exposed *Pde4b*^M220T^ mice displayed more c-Fos staining in neurons within the amygdala and hippocampal formation but less within the cortex than trauma-exposed WT mice (Fig. 6*B*), but these brain regions were not significantly different in area (number of pixels) between genotypes (Extended Data Fig. 6-1). Correlation analysis revealed a significant positive association between c-Fos staining in the dentate gyrus (DG) and the percentage of freezing over 1 min after the 10th tone–shock pairing in WT mice (*r *= 0.84; *p *< 0.05), but not in *Pde4b*^M220T^ mice (*r *= −0.14; *p *= 0.8; Fig. 6*C*). The c-Fos staining in WT mice showed significant positive correlations between subfields of the hippocampal formation (CA1–CA2 and CA2–DG) and between the CA3 and medial prefrontal cortex (mPFC), whereas *Pde4b*^M220T^ mice showed a more complex pattern of correlations between brain regions, with only the positive CA1–CA2 correlation in common with WT mice (Fig. 6*D*; Extended Data Fig. 6-2). Representative images of c-Fos-positive neurons in the cortex, amygdala, and hippocampal formation in trauma-exposed mice at 90 min post-trauma are shown in Figure 7. *Pde4b*^M220T^ mice thus demonstrated an aberrant pattern of cortical hypoactivity and hyperactivity of the hippocampus and amygdala in response to trauma.

**Figure 8.**
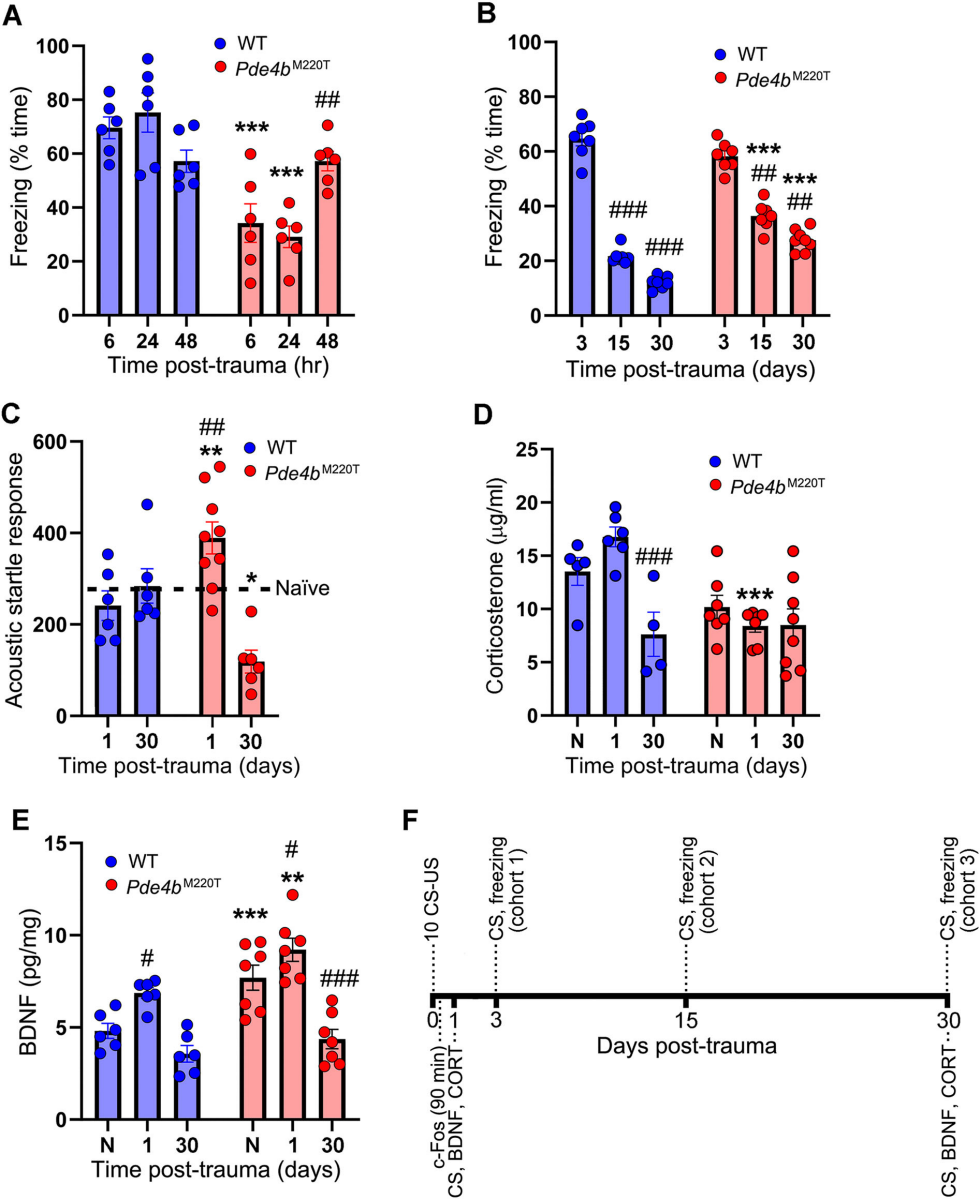
Response to trauma in *Pde4b*^M220T^ mice. ***A***, Cued fear memory test at 6, 24, and 48 h post-trauma: percentage of time spent freezing (two-way ANOVA, genotype: *F*_(1,33) _= 46.8, *p *< 0.0001; genotype × time post-trauma interaction: *F*_(2,33) _= 12.4, *p *< 0.0001). ^##^*p *< 0.01 versus *Pde4b*^M220T^ mice at 6 h post-trauma. ***B***, Cued fear memory test at 3, 15, and 30 d post-trauma: percentage of time spent freezing (two-way ANOVA, genotype: *F*_(1,36) _= 17.7, *p *< 0.001; time post-trauma: *F*_(2,36) _= 318.4, *p *< 0.0001; genotype × time post-trauma interaction: *F*_(2,36) _= 46.1, *p *< 0.0001). ^##^*p *< 0.01; ^###^*p *< 0.001 versus 3 d post-trauma within each genotype. ***C***, Amplitude of acoustic startle response to a startle stimulus (110 dB, 40 ms) at 1 and 30 d post-trauma (RM two-way ANOVA, ASR trial (1–3): *F*_(2,72) _= 15.8, *p *< 0.0001; time post-trauma: *F*_(2,36) _= 8.3, *p *< 0.001; genotype × time post-trauma interaction: *F*_(2,36) _= 13.1, *p *< 0.001). ^#^*p *< 0.05; ^##^*p *< 0.01 versus first startle pulse on the test day within each genotype. The mean startle response of naive *Pde4b*^M220T^ mice (266.3 ± 32.5; Fig. 3*A*) is indicated by a broken line. ***D***, Plasma corticosterone levels (µg/ml) in naive (nontrauma exposed) mice and in trauma-exposed mice at 1 and 30 d post-trauma (two-way ANOVA, genotype: *F*_(1,31) _= 9.65, *p *< 0.01; time post-trauma: *F*_(2,31) _= 5.89, *p *< 0.01; genotype × time post-trauma interaction: *F*_(2,31) _= 5.09, *p *< 0.05). ***p < 0.001 versus WT. ^###^*p *< 0.001 versus naive mice within each genotype. ***E***, Hippocampal BDNF levels (pg/mg total protein) in naive (nontrauma exposed) mice and in trauma-exposed mice at 1 and 30 d post-trauma (two-way ANOVA, genotype: *F*_(1,33) _= 21.2, *p *< 0.0001; time post-trauma: *F*_(2,33) _= 29.1, *p *< 0.0001; genotype × time post-trauma interaction: *F*_(2,33) _= 2.0, *p *= 0.14). ^#^*p *< 0.05; ^###^*p *< 0.001 versus naive mice within each genotype. ***F***, Timeline of cued fear memory testing of trauma-exposed mice. Top, Cued fear memory testing at 3 d (cohort 1), 15 d (cohort 2) and 30 d (cohort 3) post-trauma. Bottom, Ex vivo measurement of c-Fos at 90 min post-trauma and of BDNF and CORT 30 min after CS re-exposure at 1 and 30 d post-trauma. Data are plotted as mean ± SEM. *p < 0.05; **p < 0.01; ***p < 0.001 versus WT. BDNF, brain-derived neurotrophic factor; CORT, corticosterone; N, naive.

In cued fear memory testing, *Pde4b*^M220T^ mice froze less than WT mice at 6 and 24 h post-trauma, but at 48 h post-trauma their freezing increased to the same level as WT mice (Fig. 8*A*). To assess the response to trauma in *Pde4b*^M220T^ mice over a longer time period, separate cohorts of mice were conditioned with 10 tone–shock pairings and tested at 3, 15, or 30 d post-trauma. In a cued fear memory test at 3 d, freezing was not significantly different between genotypes. At 15 and 30 d, both genotypes displayed less freezing than at 3 d, but the level of freezing decayed more slowly in *Pde4b*^M220T^ mice (Fig. 8*B*).

Given that naive *Pde4b*^M220T^ mice displayed greater ASR than WT mice (Fig. 3*A*), we examined the effect of trauma (10 tone–shock pairings) on ASR. At 24 h post-trauma, the ASR of *Pde4b*^M220T^ mice was increased compared with WT mice (Fig. 8*C*). Comparison with naive mice indicated that trauma exposure increased the ASR in *Pde4b*^M220T^ mice (trauma-exposed: 389.5 ± 48.3; naive: 266.3 ± 32.5; *p *= 0.004) but not in WT mice (trauma-exposed: 240.5 ± 32.1; naive: 226.9 ± 31.4; *p *= 0.53). At 30 d post-trauma, the ASR of *Pde4b*^M220T^ mice had decreased to below the levels exhibited by WT mice (Fig. 8*C*).

Glucocorticoids (cortisol in humans) are the end product of the hypothalamic–pituitary–adrenal (HPA) axis that regulates the body's response to external stressors. Since individuals vulnerable for development of PTSD show low levels of circulating cortisol shortly after trauma (van Zuiden et al., 2013), we examined *Pde4b*^M220T^ mice for plasma levels of corticosterone, the primary glucocorticoid in rodents. In naive mice, plasma corticosterone levels were not significantly different between genotypes. In trauma-exposed mice, plasma corticosterone levels were significantly lower in *Pde4b*^M220T^ mice compared with WT mice at 24 h but not at 30 d post-trauma (Fig. 8*D*).

Since the cAMP signaling pathway regulates the expression of brain-derived neurotrophic factor (BDNF; Yu et al., 2021; Zhao et al., 2021), we measured hippocampal BDNF levels in mice at 24 h and 30 d post-trauma in comparison with naive mice. Naive *Pde4b*^M220T^ mice displayed increased hippocampal BDNF levels compared with naive WT mice. At 24 h post-trauma, hippocampal BDNF levels in both genotypes had increased by a similar magnitude compared with their naive counterparts, such that levels in *Pde4b*^M220T^ mice remained higher. At 30 d post-trauma, BDNF levels in both genotypes had declined to levels comparable with naive WT mice and below the levels of naive *Pde4b*^M220T^ mice (Fig. 8*E*).

## Discussion

PTSD is characterized by persistent physical and emotional reactions associated with intrusive memories that may develop in response to a traumatic event (American Psychiatric Association, 2013). Since only ∼10% of trauma-exposed individuals develop PTSD (de Vries and Olff, 2009), it is hypothesized that PTSD is associated with biological vulnerability factors already present prior to symptom onset (van Zuiden et al., 2013). Indeed, GWASs have associated *PDE4B* SNPs with PTSD (Gelernter et al., 2019; Meier et al., 2019; Nievergelt et al., 2024), as well as schizophrenia (Trubetskoy et al., 2022) and substance use disorders (Hatoum et al., 2023) that are often comorbid with it (Kessler et al., 1995). However, there is currently no evidence for functional differences between the risk and nonrisk alleles of the *PDE4B* SNPs, which makes identifying the biological basis for the genetic association extremely challenging.

We studied the effects of PDE4B missense variant M220T in transfected HEK-293 cells and male C57BL/6JJcl mice. M220T lies within the first α-helix of UCR2, so is incorporated into the long forms and short form but not the supershort form of PDE4B. The long forms dimerize, whereas the short and supershort forms are monomers (Richter and Conti, 2002; Bolger et al., 2015; Cedervall et al., 2015). PDE4B^M220T^ is present in the human genome as rs775201287, a rare coding variant (RCV) with a CADD score of 21.1 in gnomAD (Chen et al., 2024), indicating it is likely deleterious. Although the clinical significance of rs775201287 is uncertain, as sequencing studies are currently underpowered to implicate specific RCVs in psychiatric disorders (Owen et al., 2023), the largest published exome-sequencing study of schizophrenia to date identified *PDE4B*^M220T^ in two of 48,496 cases but in none of 194,644 controls (Singh et al., 2022). Thus far, a similar search for RCVs in PTSD patients has not been reported.

In contrast with the decreases in cAMP hydrolysis and DISC1 binding imparted by the Y358C variant located in the DISC1 binding site (residues 352–380) within the catalytic domain of PDE4B (McGirr et al., 2016), the M220T variant in UCR2 did not significantly alter basal PDE4B activity or binding to DISC1. However, when challenged with a forskolin-elicited increase in intracellular cAMP concentration, PDE4B1-M220T showed less cAMP hydrolysis than PDE4B1-WT, a reduction in function consistent with the subtle structural changes predicted by our molecular modeling.

In naive *Pde4b*^M220T^ mice, PDE4B protein levels were unaltered in the amygdala, as observed previously in *Pde4b*^Y258C^ mice (McGirr et al., 2016). *Pde4b* mRNA levels are decreased in the hippocampus of male C57BL/6N mice after contextual fear memory retrieval (Hori et al., 2024) and in brain tissue from rats and stress-susceptible C57BL/6NCrl mice after chronic stress (Ryan et al., 2018; Meier et al., 2019). Phospho-activation of PDE4B by PKA or Cdk5 is increased in the striatum of C57BL/6 mice after acute or chronic stress exposure (Plattner et al., 2015). A limitation of the present study is that we did not examine PDE4B expression in other brain regions associated with PTSD (Kunimatsu et al., 2020) or in trauma-exposed mice.

Behavioral phenotyping revealed that naive *Pde4b*^M220T^ mice exhibit hyperarousal or agitation in response to novelty. Patients with stress-related disorders are prone to agitation, a feeling of anxious restlessness and irritability (Taft et al., 2012). In the OF, *Pde4b*^M220T^ mice showed a preference for spending more time at the periphery over the center of the arena, suggesting an anxiety-like phenotype, but no differences were observed in time/entries in the open arms compared with closed arms in the EPM. Although both tests are used to evaluate state anxiety in mice, the OF measures inescapable open space-related anxiety, whereas the EPM measures anxiety induced by escapable open space, light/dark transitions, and elevated areas (Komada et al., 2008).

Although it is unknown whether the increased startle reactivity of PTSD patients precedes the precipitating traumatic event (Shalev et al., 2000), the startle hyperreactivity exhibited by naive *Pde4b*^M220T^ mice did not require trauma exposure. This is consistent with the enhanced ASR exhibited by rats infused with the nonsubtype-selective (pan-) PDE4 inhibitor rolipram into the amygdala (Ryan et al., 2018). *Pde4b*^M220T^ mice also exhibited deficits in PPI, similar to those reported in schizophrenia patients (Grillon et al., 1992) and inconsistently in patients with PTSD (Kohl et al., 2013; Pineles et al., 2016). During cued memory testing, *Pde4b*^M220T^ mice emitted louder fear-related calls than WT mice, demonstrating a role of PDE4B in fear communication and supporting intensity of vocalization as a biomarker of hyperarousal (Smith et al., 2023). The range of behavioral differences exhibited by *Pde4b*^M220T^ mice is consistent with the pleiotropic effects of *PDE4B* risk alleles across psychiatric diagnostic boundaries.

After exposure to trauma, *Pde4b*^M220T^ mice exhibited reduced freezing in cued fear memory testing at 6 and 24 h, but increased freezing at 48 h post-trauma, suggesting a delay in switching from an active to a passive defensive response (Fanselow, 1994). The active phase coincided with the time post-trauma when *Pde4b*^M220T^ mice exhibited increased startle and hippocampal BDNF and decreased plasma corticosterone levels. The exacerbation of startle hyperreactivity exhibited by trauma-exposed versus naive *Pde4b*^M220T^ mice is consistent with M220T conferring a pre-existing vulnerability to trauma.

The altered responses of trauma-exposed *Pde4b*^M220T^ mice may be related to altered function of the amygdala, since it mediates the potentiation of the startle reflex by fear stimuli (Davis, 2006). In support, c-Fos staining revealed that trauma-exposed *Pde4b*^M220T^ mice exhibit enhanced neuronal activity in the amygdala as well as the hippocampus. The increased amygdala activation may result from reduced inhibition by the mPFC, since trauma-exposed *Pde4b*^M220T^ mice exhibited less neuronal activity in the mPFC, as well as an aberrant pattern of correlations between neuronal activities in different brain regions. One neural circuitry model of PTSD posits that a hyperresponsive amygdala and hyporesponsive mPFC may potentially lead to deficits in extinction, emotion regulation, attention, and contextual processing (Liberzon and Sripada, 2008).

Activation of cAMP signaling promotes the expression of BDNF, the terminal downstream protein neurotrophic factor in the pathway (Yu et al., 2021; Zhao et al., 2021). As BDNF is involved in synaptic plasticity related to learning and memory (Andero and Ressler, 2012; Leal et al., 2017), our finding of elevated hippocampal BDNF levels in *Pde4b*^M220T^ mice, before and 24 h after trauma, suggests that the PDE4B^M220T^ variant facilitates hippocampal synaptic plasticity. However, a limitation of this study is that we did not examine proteins upstream of BDNF in the cAMP signaling pathway in *Pde4b*^M220T^ mice. Facilitation of hippocampal function in *Pde4b*^M220T^ mice is supported by our observations of enhanced neuronal activity and enhanced performance in hippocampus-dependent spatial memory tests. While the behavior of *Pde4b*^M220T^ mice in the OLT may have been influenced by their altered response to novelty, supportive evidence in the literature associates PDE4B with cognitive function (McGirr et al., 2016; Gurney, 2019; Armstrong et al., 2024).

The freezing of a separate cohort of trauma-exposed mice in cued fear memory testing at 3 d was similar to the levels at 48 h post-trauma (∼60%) in both genotypes. The slower decay in freezing exhibited by other cohorts of *Pde4b*^M220T^ mice at 15 and 30 d post-trauma demonstrates enhanced persistence of traumatic memories and exaggerated fear responses. PTSD patients similarly exhibit persistence of the traumatic memory for months, years, or decades after the traumatic event. Failure in extinction or abnormal reconsolidation has been proposed as memory processes by which fear conditioning could lead to persistence of the traumatic memory over time (Careaga et al., 2016). Since both phenomena depend on memory retrieval (Kida, 2019), a limitation of our experimental design (Fig. 7*F*), without repeated re-exposure to the CS, is that it provides no information on whether the memory persistence of *Pde4b*^M220T^ mice is due to deficient fear extinction. It is also a limitation that we did not include female mice and *Pde4b*^M220T^ heterozygotes, since the prevalence of PTSD is twice as high in women versus men (Kessler et al., 1995) and all 21 human carriers of *PDE4B*^M220T^ in gnomAD are heterozygous (Chen et al., 2024).

Based on GWAS data, PDE4B-selective inhibitors have been suggested as potential treatments for stress-related disorders (Meier et al., 2019). The PDE4B-selective inhibitor A-33 has shown antidepressant-like effects in male Hsd:ICR mice in the FST (Zhang et al., 2017). However, to date, testing in PTSD-relevant models is restricted to pan-PDE4 inhibitors, which have limited clinical utility owing to dose-dependent side effects of nausea and emesis, attributed to inhibition of PDE4D (Giembycz, 2002). Administration of rolipram 15 min before contextual fear memory testing, 24 h postconditioning, was shown to decrease contextual freezing and increase fear extinction over 15 min in male C57BL/6J mice that were exposed to trauma 14 d earlier (Gao et al., 2023).

In summation, GWASs have associated variants in *PDE4B* with PTSD and other psychiatric disorders comorbid with it, but the pathophysiological mechanisms of genetic risk involving *PDE4B* are poorly understood. *Pde4b*^M220T^ mice replicating human variant rs775201287 exhibit PTSD-relevant neural and behavioral phenotypes that are exacerbated by trauma, thus highlighting how genetic variation of PDE4B may contribute to PTSD risk and suggesting a potential route to treatment.
