# Supplementary material for: PDE4B Missense Variant Increases Susceptibility to Post-traumatic Stress Disorder-Relevant Phenotypes in Mice
Source: J Neurosci. 2024 Sep 10;44(43):e0137242024. doi: 10.1523/JNEUROSCI.0137-24.2024 (PMC11502227; doi:10.1523/JNEUROSCI.0137-24.2024)
Supplement: Figure 2-1 — Behavior of Pde4bM220T and WT mice in the elevated plus maze. Pde4bM220T mice made more entries to the open arms, closed arms and center, and more passages between the open arms, than WT mice. *p < 0.05, **p < 0.01, ***p < 0.001 versus WT. ANOVA, analysis of variance; No., number of; WT, wild-type. Download Figure 2-1, DOCX file. [file jneuro-44-e0137242024-s002.docx]

| Parameter | Genotype | | ANOVA |
| --- | --- | --- | --- |
|  | WT  (*n* = 8) | *Pde4b*^M220T^  (*n* = 8) |  |
| N^o.^ entries to open arms | 3.6 ± 1.7 | 9.6 ± 1.9* | *F*_(1,14)_ = 8.1, *p* < 0.05 |
| N^o.^ entries to center | 22.8 ± 2.4 | 34.3 ± 2.3*** | *F*_(1,14)_ = 17.3, *p* < 0.001 |
| N^o.^ entries to closed arms | 19.2 ± 1.8 | 24.3 ± 1.7* | *F*_(1,14)_ = 7.3, *p* < 0.05 |
| N^o^. passages between open arms | 2.3 ± 0.8 | 6.0 ± 1.2* | *F*_(1,14)_ = 6.3, *p* < 0.05 |
| N^o.^ passages between closed arms | 13.6 ± 1.8 | 13.4 ± 1.2 | *F*_(1,14)_ = 0.01, *p* > 0.05 |

**Figure 2-1.** Behavior of *Pde4b*^M220T^ and WT mice in the elevated plus maze. *Pde4b*^M220T^ mice made more entries to the open arms, closed arms and center, and more passages between the open arms, than WT mice. **p* < 0.05, ***p* < 0.01, ****p* < 0.001 versus WT. ANOVA, analysis of variance; N^o.^, number of; WT, wild-type.
