# Supplementary material for: PDE4B Missense Variant Increases Susceptibility to Post-traumatic Stress Disorder-Relevant Phenotypes in Mice
Source: J Neurosci. 2024 Sep 10;44(43):e0137242024. doi: 10.1523/JNEUROSCI.0137-24.2024 (PMC11502227; doi:10.1523/JNEUROSCI.0137-24.2024)
Supplement: Figure 2-2 — Behavior of Pde4bM220T and WT mice in the three-chamber social approach test. Pde4bM220T mice did not display differences in habituation, sociability or social memory. No., number of; WT, wild-type. Download Figure 2-2, DOCX file. [file jneuro-44-e0137242024-s003.docx]

| Parameter | WT (*n* = 16) | *Pde4b*^M220T^ (*n* = 11) |
| --- | --- | --- |
| *Habituation* |  |  |
| Time in left chamber (s) | 99.7 ± 5.6 | 102.7 ± 6.8 |
| Time in right chamber (s) | 104.3 ± 9.3 | 107.1 ± 8.1 |
| *Sociability* |  |  |
| N^o.^ entries to empty side | 24.9 ± 1.5 | 23.6 ± 1.9 |
| N^o.^ entries to stranger 1 side | 31.3 ± 1.4 | 35.9 ± 1.5 |
| *Social memory* |  |  |
| N^o.^ entries to stranger 1 side | 17.3 ± 0.8 | 15.9 ± 1.3 |
| N^o.^ entries to stranger 2 side | 20.1 ± 1.1 | 21.6 ± 1.6 |

**Figure 2-2.** Behavior of *Pde4b*^M220T^ and WT mice in the three-chamber social approach test. *Pde4b*^M220T^ mice did not display differences in habituation, sociability or social memory. N^o.^, number of; WT, wild-type.
