# Supplementary material for: PDE4B Missense Variant Increases Susceptibility to Post-traumatic Stress Disorder-Relevant Phenotypes in Mice
Source: J Neurosci. 2024 Sep 10;44(43):e0137242024. doi: 10.1523/JNEUROSCI.0137-24.2024 (PMC11502227; doi:10.1523/JNEUROSCI.0137-24.2024)
Supplement: Figure 4-1 — Behavior of Pde4bM220T and WT mice in the object location test. Times spent freezing and exploring all objects were not significantly different between genotypes. WT, wild-type. Download Figure 4-1, DOCX file. [file jneuro-44-e0137242024-s004.docx]

| Parameter | WT (*n* = 7–9) | *Pde4b*^M220T^ (*n* = 7–9) |
| --- | --- | --- |
| *5 min training* |  |  |
| Freezing (s) | 10.5 ± 3.6 | 11.6 ± 2.3 |
| Time exploring all objects (s) | 150.3 ± 8.9 | 142.8 ± 12.8 |
| *15 min training* |  |  |
| Freezing (s) | 21.7 ± 8.4 | 22.5 ± 7.2 |
| Time exploring all objects (s) | 458.2 ± 15.7 | 512.7 ± 23.5 |

**Figure 4-1.** Behavior of *Pde4b*^M220T^ and WT mice in the object location test. Times spent freezing and exploring all objects were not significantly different between genotypes. WT, wild-type.
