# Supplementary material for: PDE4B Missense Variant Increases Susceptibility to Post-traumatic Stress Disorder-Relevant Phenotypes in Mice
Source: J Neurosci. 2024 Sep 10;44(43):e0137242024. doi: 10.1523/JNEUROSCI.0137-24.2024 (PMC11502227; doi:10.1523/JNEUROSCI.0137-24.2024)
Supplement: Figure 6-1 — Area of each brain region examined for c-Fos-positive neurons in Pde4bM220T and WT mice at 90 min post-trauma. Brain regions in the cortex, amygdala and hippocampal formation were not significantly different in area (number of pixels) between genotypes. WT, wild-type. Download Figure 6-1, DOCX file. [file jneuro-44-e0137242024-s005.docx]

| Region of interest | Number of pixels | | ANOVA |
| --- | --- | --- | --- |
|  | WT (*n* = 7 mice)  (*n*=4-5 sections/mouse) | *Pde4b*^M220T^ (*n* = 6 mice)  (*n*=4-5 sections/mouse) |  |
| *Cortex* |  |  |  |
| Medial prefrontal cortex | 191795.9 ± 4949.5 | 188595.0 ± 4358.6 | *F*_(1,52)_ = 0.2, *p* > 0.05 |
| Piriform cortex | 68761.3 ± 5031.4 | 59664.6 ± 7609.0 | *F*_(1,80)_ = 0.1, *p* > 0.05 |
| *Amygdala* |  |  |  |
| Basolateral amygdala | 101864.7 ± 9557.3 | 113671.2 ± 10759.4 | *F*_(1,76)_ = 1.2, *p* > 0.05 |
| Basomedial amygdala | 120939.9 ± 9625.2 | 124934.9 ± 7174.7 | *F*_(1,6)_ = 0.8, *p* > 0.05 |
| *Hippocampal formation* |  |  |  |
| CA1 | 29260.7 ± 1968.1 | 33641.4 ± 2321.7 | *F*_(1,58)_ = 1.7, *p* > 0.05 |
| CA2 | 57343.7 ± 4471.6 | 48386.0 ± 2442.4 | *F*_(1,53)_ = 3.6, *p* > 0.05 |
| CA3 | 61449.4 ± 2923.6 | 58379.8 ± 3413.2 | *F*_(1,56)_ = 0.4, *p* > 0.05 |
| Dentate gyrus | 97050.0 ± 6005.7 | 94613.85 ± 3175.5 | *F*_(1,45)_ = 0.1, *p* > 0.05 |

**Figure 6-1.** Area of each brain region examined for c-Fos-positive neurons in *Pde4b*^M220T^ and WT mice at 90 min post-trauma. Brain regions in the cortex, amygdala and hippocampal formation were not significantly different in area (number of pixels) between genotypes. WT, wild-type.
