# Supplementary material for: PDE4B Missense Variant Increases Susceptibility to Post-traumatic Stress Disorder-Relevant Phenotypes in Mice
Source: J Neurosci. 2024 Sep 10;44(43):e0137242024. doi: 10.1523/JNEUROSCI.0137-24.2024 (PMC11502227; doi:10.1523/JNEUROSCI.0137-24.2024)
Supplement: Figure 6-2 — Pearson’s correlation coefficients (r) between c-Fos staining in different brain regions in Pde4bM220T and WT mice at 90 min post-trauma. In WT mice, the c-Fos staining showed significant positive correlations between subfields of the hippocampal formation (CA1–CA2 and CA2–DG) and between the CA3 and mPFC. In Pde4bM220T mice, there was a more complex pattern of correlations between brain regions, with only the positive CA1–CA2 correlation in common with WT mice. Bold text, significant correlation; red text, common between WT and Pde4bM220T mice; n = 4-5 sections/mouse. Download Figure 6-2, DOCX file. [file jneuro-44-e0137242024-s006.docx]

**WT (*n* = 7 mice)**

|  | mPFC | PC | BLA | BMA | CA1 | CA2 | CA3 |
| --- | --- | --- | --- | --- | --- | --- | --- |
| PC | *r* = 0.43  *p* = 0.07 |  |  |  |  |  |  |
| BLA | *r* = 0.36  *p* = 0.14 | *r* = 0.29  *p* = 0.24 |  |  |  |  |  |
| BMA | *r* = 0.14  *p* = 0.59 | *r* = 0.31  *p* = 0.20 | *r* = 0.45  *p* = 0.06 |  |  |  |  |
| CA1 | *r* = 0.39  *p* = 0.17 | *r* = 0.13  *p* = 0.62 | *r* = 0.43  *p* = 0.07 | *r* = 0.25  *p* = 0.31 |  |  |  |
| CA2 | *r* = 0.28  *p* = 0.26 | *r* = 0.21  *p* = 0.41 | *r* = 0.10  *p* = 0.07 | *r* = 0.31  *p* = 0.22 | ***r* = 0.59**  ***p* = 0.01** |  |  |
| CA3 | ***r* = 0.61**  ***p* = 0.007** | *r* = 0.03  *p* = 0.91 | *r* = 0.38  *p* = 0.12 | *r* = -0.28  *p* = 0.27 | *r* = 0.38  *p* = 0.13 | *r* = 0.12  *p* = 0.62 |  |
| DG | *r* = 0.29  *p* = 0.24 | *r* = -0.07  *p* = 0.79 | *r* = 0.02  *p* = 0.95 | *r* = 0.32  *p* = 0.19 | *r* = 0.46  *p* = 0.053 | ***r* = 0.66**  ***p* = 0.003** | *r* = 0.15  *p* = 0.55 |

***Pde4b*^M220T^ (*n* = 6 mice)**

|  | mPFC | PC | BLA | BMA | CA1 | CA2 | CA3 |
| --- | --- | --- | --- | --- | --- | --- | --- |
| PC | ***r* = 0.46**  ***p* = 0.02** |  |  |  |  |  |  |
| BLA | *r* = -0.26  *p* = 0.18 | *r* = -0.06  *p* = 0.77 |  |  |  |  |  |
| BMA | *r* = 0.26  *p* = 0.18 | ***r* = 0.39**  ***p* = 0.047** | *r* = 0.22  *p* = 0.26 |  |  |  |  |
| CA1 | ***r* = -0.49**  ***p* = 0.008** | ***r* = -0.52**  ***p* = 0.006** | *r* = -0.22  *p* = 0.27 | *r* = -0.48  *p* = 0.01 |  |  |  |
| CA2 | ***r* = -0.55**  ***p* = 0.003** | ***r* = -0.49**  ***p* = 0.010** | *r* = -0.24  *p* = 0.27 | ***r* = -0.57**  ***p* = 0.002** | ***r* = 0.69**  ***p*= 0.001** |  |  |
| CA3 | *r* = 0.05  *p* = 0.81 | *r* = -0.34  *p* = 0.08 | *r* = -0.35  *p* = 0.07 | ***r* = -0.49**  ***p* = 0.009** | ***r* = 0.49**  ***p* = 0.01** | ***r* = 0.49**  ***p* = 0.009** |  |
| DG | *r* = 0.31  *p* = 0.12 | *r* = -0.16  *p* = 0.42 | ***r* = -0.55**  ***p* = 0.003** | *r* = -0.17  *p* = 0.40 | *r* = 0.13  *p* = 0.51 | *r* = 0.12  *p* = 0.55 | ***r* = 0.56**  ***p* = 0.003** |

**Figure 6-2.** Pearson’s correlation coefficients (*r*) between c-Fos staining in different brain regions in *Pde4b*^M220T^ and WT mice at 90 min post-trauma. In WT mice, the c-Fos staining showed significant positive correlations between subfields of the hippocampal formation (CA1–CA2 and CA2–DG) and between the CA3 and mPFC. In *Pde4b*^M220T^ mice, there was a more complex pattern of correlations between brain regions, with only the positive CA1–CA2 correlation in common with WT mice. Bold text, significant correlation; red text, common between WT and *Pde4b*^M220T^ mice; *n*=4-5 sections/mouse.
